# Predictors of Elevated Defibrillation Threshold with the Subcutaneous Implantable Cardioverter-defibrillator

**DOI:** 10.19102/icrm.2017.081203

**Published:** 2017-12-15

**Authors:** Khuyen Do, Philip Chang, Tomas Konecny, Steven K. Carlson, Han Tun, Mary Huntsinger, Rahul N. Doshi

**Affiliations:** ^1^Keck USC Medical Center and LAC-USC Medical Center, Keck School of Medicine of USC, Los Angeles, California, USA

**Keywords:** Body mass index, defibrillation threshold testing, subcutaneous implantable cardioverter-defibrillator, obesity, end-stage renal disease

## Abstract

There are limited data regarding defibrillation thresholds (DFTs) for the subcutaneous implantable cardioverter-defibrillator (S-ICD), and factors associated with elevated DFTs remain incompletely understood. The objective of this study was to determine the factors associated with elevated DFTs in patients undergoing S-ICD implantation. A retrospective cross-sectional analysis of all patients undergoing S-ICD implantation at our institution between 2013 and 2016 who underwent step-down DFT testing was performed. Factors associated with a higher DFT were analyzed. In total, 56 patients (mean age: 49.3 ± 13.1 years, mean left ventricular ejection rate: 31.1% ± 13.7%) underwent S-ICD implantation in the study period. Full DFT testing was performed in 31 of the 56 patients (55%), with an average DFT of 46.4 joules (J) ± 25.9 J found among this cohort. The DFT was > 65 J in five of the 31 patients (16%). A high DFT was associated with increased body mass index (BMI) (37.7 kg/m^2^ versus 29.4 kg/m^2^; p = 0.02) and either increased septal or posterior wall thickness (1.5 cm versus 1.0 cm; p = 0.0003 and 1.4 cm versus 1.1 cm; p= 0.003, respectively). Patients with high DFTs also had higher failed shock impedance values (138 *Ω* versus 71 *Ω*; p = 0.005). Renal failure did not appear to affect DFT (51.4 J versus 51.7 J; p = 0.99). BMI, body surface area (BSA), and septal and posterior left ventricular wall thickness predicted elevated DFT on univariate analysis, although findings were not significant with multivariate analysis due to the small sample size. Thus, elevated S-ICD DFT appears to be associated with increased BMI, BSA, and septal or posterior wall thickness. In contrast, dialysis-dependent renal failure is not associated with elevated DFT. Further investigation is necessary in order to better characterize and predict which patients are at-risk for high DFTs.

## Introduction

Implantable cardioverter-defibrillators (ICDs) have been shown to improve survival in patients at high-risk for sudden arrhythmic death for more than 30 years.^1^ The survival benefits from ICDs stem from their ability to acutely terminate ventricular arrhythmias (VAs) using high-energy shocks. While shock efficacy remains probabilistic, increased confidence in an ICD’s ability to successfully terminate spontaneous VAs can be established with defibrillation threshold (DFT) testing.^2^ The practice of routine DFT testing at the time of implant has decreased over the years, owing to advancements in ICD technology including improved lead design and higher shock capacity. Several recent randomized trials have shown equivalent clinical outcomes with or without DFT testing at the time of initial transvenous ICD implantation across multiple cardiac substrates.^3^ These results have led to changes in practice recommendations that reserve routine DFT testing to selected populations at initial transvenous ICD implant and for patients with atypical implant configurations.

Experience with transvenous ICD systems across multiple cardiac substrates and patient populations have retrospectively elucidated factors that may be associated with higher DFTs. These factors include reduced ejection fraction, non-ischemic cardiomyopathy, high left ventricular mass, male gender, amiodarone use, and increased ventricular wall thickness. However, the associations of these factors vary across different studies and have not been reproducible prospectively.^4^ Similar studies, long-term experiences, and variables predicting high DFTs with subcutaneous ICD (S-ICD) use remain lacking.

Given the limited but growing experience with S-ICD use, functional defibrillation testing is still recommended, though formal step-down DFT testing is not required at implant.^5^ In the pilot S-ICD study, the average energy required to terminate induced ventricular fibrillation was 36.6 J.^6^ In this study, we sought to evaluate the spectrum of true DFTs across a diverse group of patients implanted with the S-ICD and to identify potential factors that may predict elevated DFT.

## Methods

This was a retrospective, non-randomized, cross-sectional study of all patients receiving the S-ICD at our institution from 2013 to 2016. The study was approved by our local institutional review board, which waived the need for patient consent due to the study’s design of a retrospective nature with patient data de-identified.

### Study population and S-ICD implantation

The study population included all patients aged > 18 years old who met the criteria for primary or secondary prevention S-ICD implantation. All patients were considered candidates for the S-ICD based on a lack of primary pacing indications, concomitant epicardial or unipolar pacing systems, and documented VA that could be terminated with anti-tachycardia pacing (ATP). All patients had pre-procedural surface electrocardiogram (ECG) screening that confirmed adequate R-wave to T-wave ratios in at least one of three conventional vectors. Medical history, demographic data, clinical risk factors, current medication usage, etiology of cardiomyopathy, ECG data, echocardiographic measurements, basic laboratory data, and indications for ICD implantation were recorded for each individual. All S-ICD implants were performed by one of three implanting physicians. All procedures were performed in the electrophysiology laboratory with general anesthesia and endotracheal intubation for all patients. The use of fluoroscopy during electrode insertion and either a two- or three-incision approach to device implantation were left up to the discretion of the implanting physician. The device generator location was the left lateral chest wall for all patients with initial electrode positioning in either the left or right parasternal locations, depending on preprocedural screening evaluation results.

### Defibrillation threshold testing protocol

A fluoroscopic image of the chest was obtained following implant completion but prior to DFT testing to confirm appropriate electrode and generator positioning. Basic device interrogation confirmed acceptable sensed electrograms during baseline rhythm. The incision and pocket sites were irrigated and massaged for de-airing and single-layer suture closure of the incisions was performed. All patients were intubated and the phase of respiration was not controlled during shocks. Step-down DFT testing was then performed by inducing VF through the device using a 50 Hz transthoracic current. The initial shock energy output from the device was typically set at 65 J, but was able to be altered at the discretion of the implanting physician. If any shock failed to convert VF, a second shock at maximum (ie, 80 J) output was delivered through the device. External defibrillation was immediately available for rescue if S-ICD shocks failed to terminate VF. If the first shock was successful, a brief waiting period of at least three minutes was observed, followed by reinduction of VF and attempted defibrillation with a shock output of 10 joules (J) or 15 J below the previous shock. This step-down process was repeated until the lowest level of energy that failed to defibrillate was reached.

The DFT was defined as the lowest level of energy that successfully terminated VF. Given that the lowest programmable shock output from the device was 10 J, patients who successfully converted at this value were classified as having a DFT ≤ 10 J. Similarly, failure to convert at the maximum 80 J delivery led to the definition of DFT > 80 J. Patients with a failed shock at 80 J were assigned a DFT of 100 J in accordance with the pilot S-ICD study.^6^

Full DFT testing was defined as step-down testing that reached the DFT or successful conversion at the lowest deliverable energy output (10 J). Partial DFT testing was defined as step-down testing with successful shocks < 65 J and > 10 J without observation of defibrillation failure. Functional defibrillation testing was defined as two successful shocks in standard or reverse polarity at 65 J without subsequent testing at any lower energy output. The decision to perform full, partial, functional, or no defibrillation testing was made at the discretion of the implanting physician. Patients with a failed shock of > 65 J were classified as having a high DFT. All patients with DFT ≤ 65 J were classified as having an acceptable DFT.

### Clinical and laboratory data

Demographic, clinical, pharmacologic, and laboratory data were collected from thorough health record review from each patient’s index hospitalization or outpatient visit where S-ICD implantation was performed. Laboratory data obtained on the day of the procedure, or the latest values available prior to the procedure if the former were not available, were included. The etiology of cardiomyopathy was determined based on clinical documentation. Body mass index (BMI) and body surface area (BSA) were calculated at the time of implant.

### ECG and echocardiogram parameters

All available 12-lead ECGs taken at or before S-ICD implantation were reviewed. The amplitude of the QRS was recorded from the lateral leads (ie, I, aVL, V5, V6). Patients were classified as having left ventricular hypertrophy (LVH) by ECG if they met the Sokolow-Lyon or Cornell criteria.

Available echocardiographic measurements including left ventricular ejection fraction (LVEF), septal and posterior wall thicknesses, left ventricular end-diastolic dimension (LVEDD) measured in the parasternal long axis, and the presence or absence of pericardial effusion, were reviewed and included in the study analysis.

### Statistical methods

Statistical analysis was performed using SAS version 9.4 (SAS Institute, Cary, NC, USA). Categorical values are presented as numbers and percentages. Continuous variables are presented as means with standard deviations. A Student’s t-test was used to compare categorical values, and a chi-square or Fisher’s exact test was used to compare continuous variables. A p-value < 0.05 was considered to be statistically significant. Uni- and multivariable analyses were incorporated to further evaluate the association between different factors and high DFT.

## Results

**Figure 1:**
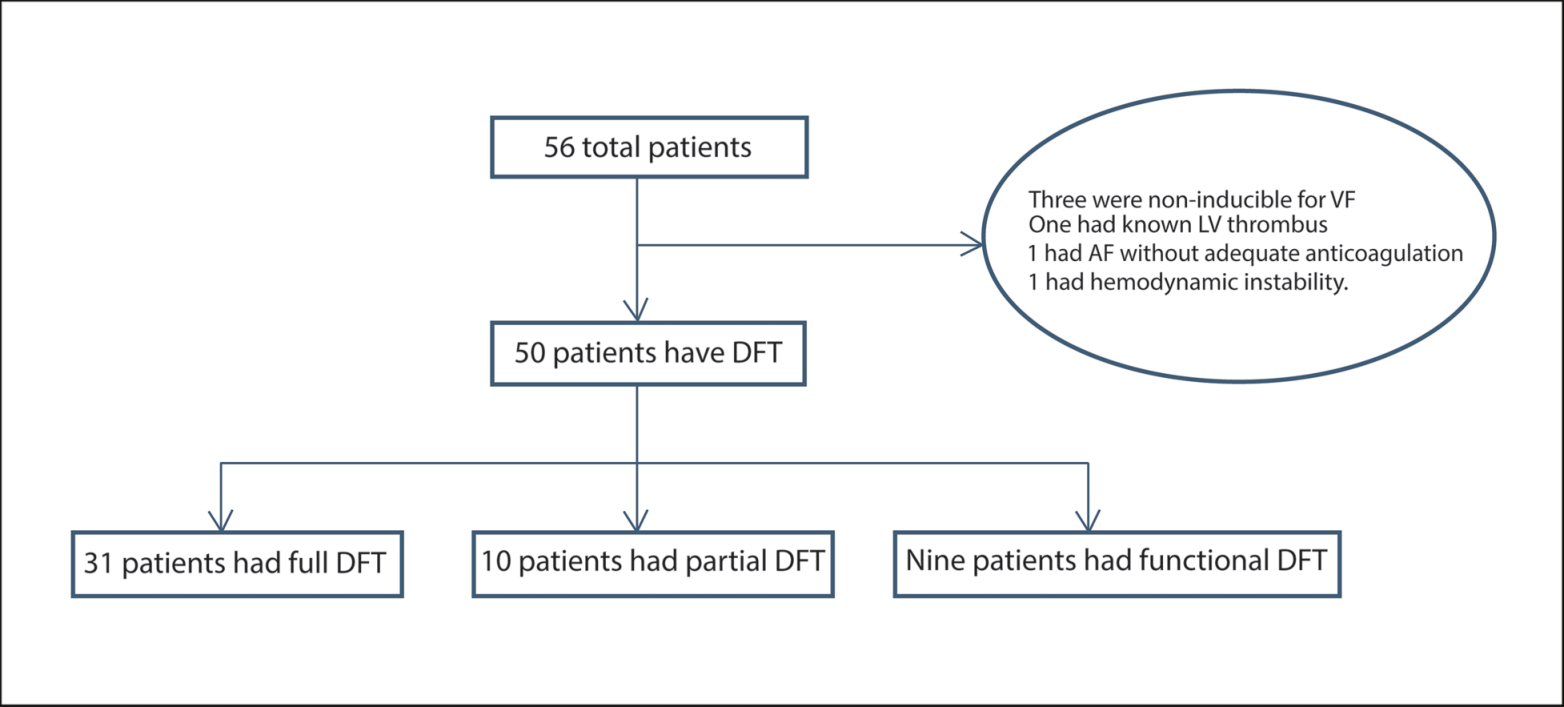
Flow diagram of all patients in the study. DFT: defibrillation threshold; VF: ventricular fibrillation; LV: left ventricle.

A total of 56 patients underwent S-ICD implantation over the study period. The baseline demographic characteristics of the 50 patients who underwent DFT testing are listed in **Table 1**. The mean age of the study population was 50.9 ± 13.8 years, and the average LVEF was 31.4% ± 13.4%. Forty-two percent of patients (21 patients) had ischemic cardiomyopathy. Among the study cohort, a total of 16 patients (32%) had end-stage renal disease (ESRD) and were on hemodialysis (HD). A total of 14 patients (28%) were implanted for secondary prevention. Some variation of defibrillation testing was performed in 50 of the 56 patients (89%): full DFT testing was performed in 31 (56%), partial DFT testing was performed in 10 (18%), and functional defibrillation testing was performed in nine (16%) **(Figure 1)**. The average number of shocks during testing was 3.9 ± 2.1, and the average DFT for the entire cohort was 50.7 J ± 26.3 J. There were no significant differences in terms of demographic, clinical, or pharmacological variables among the testing subgroups. The reasons provided for abstinence from testing in cases in which testing was not performed included an inability to induce sustained VF (three patients), known left ventricular thrombus (one patient), the presence of atrial fibrillation with inadequate anticoagulation (one patient), and the presence of hemodynamic instability during implantation (one patient). In the one case of hemodynamic instability, profound bradycardia and hypotension developed during device implantation without a clear cause, requiring chest compressions to be performed briefly, with a prompt resolution ultimately achieved.

### Full step-down DFT testing

Thirty-one patients underwent full step-down DFT testing. Five patients had high DFT while 26 patients had normal DFT **(Table 2)**. The overall average DFT was 46.4 J ± 25.9 J. The average DFT was 88.0 J ± 10.9 J for the high DFT group and 38.5 J ± 19.4 J for the normal DFT group (p = 0.0005). The average impedance for a failed shock was higher in the high DFT group as compared with in the normal DFT group (138 Ω versus 71.5 Ω; p = – 0.005). The average number of shocks delivered during testing was similar between the high and normal DFT groups (5.8 versus 4.7; p = 0.23).

Among the five patients with a shock failure > 65 J, three were started on oral sotalol for an average of 14 days and were referred for repeat defibrillation testing. For these individuals, repeat DFT testing was successful at 50 J in one patient and at 40 J in another, while VF was non-inducible in the last patient. One patient with high DFT at device implantation refused to undergo repeat DFT testing, and one patient was not referred for repeat DFT testing.

No other significant demographic differences were noted between the high and normal DFT groups. The etiology of cardiomyopathy and the prevalence of β-blocker and amiodarone use were similar in both groups.

### Obesity

The average BMI among the 31 patients who underwent full DFT testing was 30.7 ± 6.9 kg/m^2^. However, the average BMI of those patients with high DFT was significantly higher than that of the normal DFT group (37.7 kg/m^2^ versus 29.4 kg/m^2^; p = 0.02). All patients with a higher DFT had a BMI > 30 kg/m^2^. A significantly higher percentage of patients with high DFTs had BMI > 35 kg/m^2^ (60% versus 11.5%; p = 0.04) as compared with those with normal DFTs. The average BSA was also higher in the high DFT group than in the normal DFT group (2.4 m^2^ versus 1.9 m^2^; p = 0.0002).

**Figure 2:**
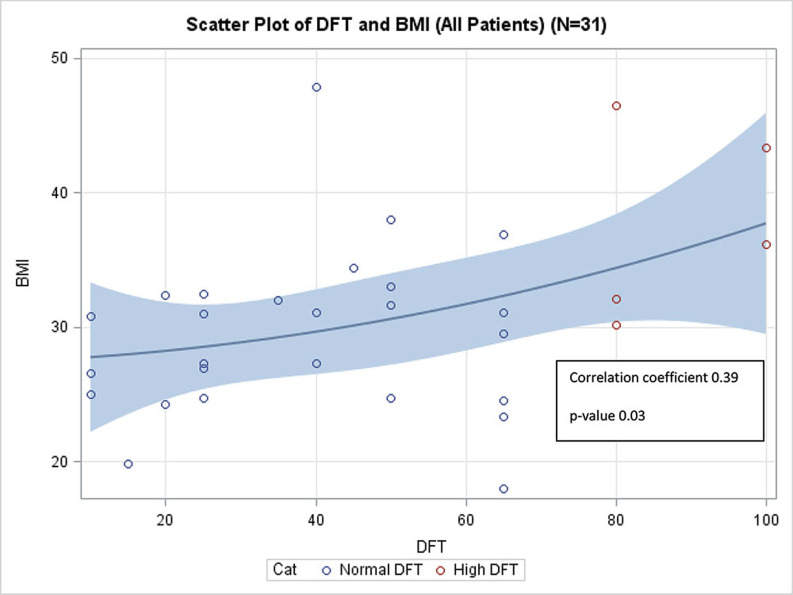
Correlation between BMI and DFT. Higher BMI was associated with increased DFT. DFT: defibrillation threshold; BMI: body mass index.

**Figure 3:**
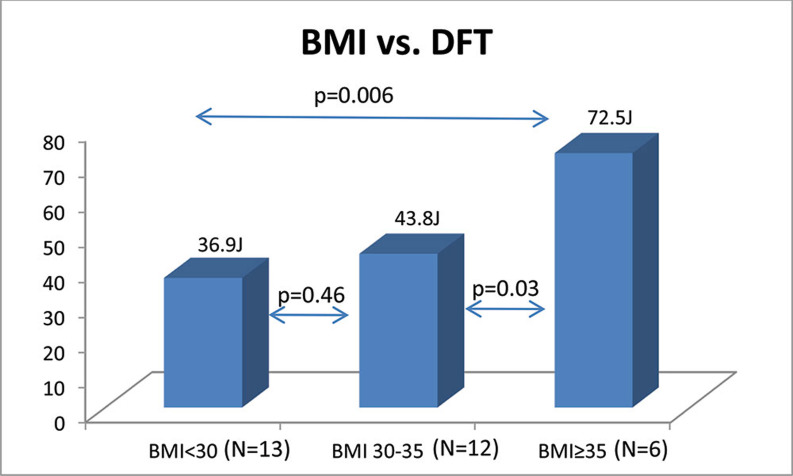
Average DFT with respect to the different BMI groups. Patients with higher BMIs had significantly higher average DFTs. DFT: defibrillation threshold, BMI: body mass index (kg/m^2^); J: joules.

A positive correlation between increasing BMI and DFT was present (p = 0.03) **(Figure 2)**, with an average DFT of 36.9 J for patients with a BMI of < 30 kg/m^2^ 43.8 J for patients with a BMI of 30 kg/m^2^ to 35 kg/m^2^ and 72.5 J for patients with a BMI of ≥ 35 kg/m^2^ (p = 0.006), respectively. Patients with a BMI of ≥ 35 kg/m^2^ had significantly higher DFTs than either those with a BMI of < 30 kg/m^2^ (p=0.006) or those with a BMI of 30 kg/m^2^ to 35 kg/m^2^ (p=0.03), respectively **(Figure 3)**. There was no significant difference in DFT between patients with a BMI of < 30 kg/m^2^ and those with a BMI of 30 kg/m^2^ to 35 kg/m^2^ (p = 0.46).

### Echocardiographic and ECG measurements

Patients in the high DFT group had significantly higher septal (1.5 cm versus 1.0 cm; p = 0.0003) and posterior (1.4 cm versus 1.1 cm; p = 0.003) wall thicknesses than patients in the normal DFT group. The average ejection fraction was not significantly different between the normal and high DFT groups (33.6% versus 25%; p = 0.28) and the presence of pericardial effusion was also similar in both.

Additionally, the incidence of LVH on ECG was similar between the normal and high DFT groups, and average voltages in the lateral leads were similar **(Table 3)**.

### End-stage renal disease

Six of the 31 patients (19%) who underwent full DFT testing had ESRD, who were patients who were actively receiving hemodialysis. **Table 4** shows the baseline characteristics of patients with and without ESRD. Those with ESRD had significantly higher incidence of ischemic cardiomyopathy (83.3% versus 28%; p = 0.02). There were no significant differences in the indication for S-ICD, ECG measurements, or echocardiographic measurements. Average DFT (51.7 J versus 51.4 J; p = 0.99) and the average number of shocks during testing (5.3 versus 4.8; p = 0.52) were similar between patients with and without ESRD. The average shock impedances were higher in patients without ESRD than in those with ESRD, both with respect to successful shocks (82.9 J versus 55.2 J; p = 0.03) and failed shocks (97.9 J versus 56.3 J, p = 0.04). The average BMI in patients with ESRD was lower than in those without, but not in a statistically significant manner (26.9 kg/m^2^ versus 31.7 kg/m^2^; p = 0.13).

### Adverse events related with defibrillation testing

All inducible VF episodes were appropriately detected by the S-ICD without dropout or under-sensing. There were no complications attributable to DFT testing, including absence of stroke, myocardial infarction, pulmonary embolism, or death. Two patients experienced bradycardia and hypotension at the end of DFT testing, both requiring short-term pharmacologic intervention and monitoring in the intensive care unit post-procedure. One patient experienced left shoulder dislocation secondary to inadequate arm bracing during DFT testing, which was successfully reduced by closed reduction following the procedure. There were no instances of infection following S-ICD implantation in this study, and no long-term adverse outcomes that could be attributed to DFT testing were observed.

### Univariate analyses

**Table 5** shows the results of univariate logistic regression analysis. BMI, BSA, and septal and posterior wall thicknesses were significant predictors of high DFT (p < 0.05). High failed shock impedance (p = 0.05) did not meet a level of statistical significance to predict high DFT. Owing to our small patient sample size, no factor was singled out as a significant predictor of high DFT in multivariate analysis (data not shown).

## Discussion

To our knowledge, this is the first study to examine step-down DFT testing in S-ICD patients since the completion of the pilot S-ICD study. In that study, the average DFT was 36.6 J among a limited and selected group of patients. Data regarding average DFTs in larger and more diverse populations undergoing S-ICD implantation are lacking. High shock efficacy at implantation based only on the successful termination of two consecutive VF inductions with 65-J shocks was reported as > 95% efficacious in the EFFORTLESS Registry and pooled experience.^7^ Data from the National Cardiovascular Data Registry for S-ICD implantation reports that defibrillation testing at the time of implant has declined to be completed in only 75% of all procedures. However, there has been an increasing number of reports of failed S-ICD defibrillation at 65 J when testing is performed.^8^ These recent findings show a concerning trend of higher DFT with reduced defibrillation testing at the time of implantation.

Published results from the EFFORTLESS Registry and pooled European and United States experience have reported an average patient BMI of 28.2 ± 5 kg/m^2^. In comparison, the members of our study cohort who underwent full step-down testing had a higher average BMI (30.7 ± 6.9 kg/m^2^), and there were more subjects with extremely elevated BMI > 40 kg/m^2^ (three patients versus none) and more with DFT values of 80 J or higher (five patients versus one). Additionally, the average DFT was 46.4 J for all patients who underwent full step-down DFT testing. If we exclude the high DFT group, however, the average DFT was 38.5 J, which is similar to the 36.6 J average in the pilot S-ICD study. Of note, there were two patients (6.4%) with DFT > 80 J in our study population as compared with only one patient (2%) in the pilot study.^6^ The incidence of DFT > 65 J was 16% in our study as compared with 7.2% in the US Registry.^8^ Interestingly, this incidence increased from 5.3% to 11% over time in the US S-ICD registry, which may be related to the inclusion of a more heterogeneous population of patients and cardiac substrates receiving S-ICDs as well as selection bias in deciding who undergoes DFT testing.

Both suboptimal device position and lead impedance have been associated with higher DFTs on computer modeling.^9^ Frommeyer et al. looked at safety margin DFT testing at the time of implant in 102 patients and noted a 6.1% incidence of failed shocks at maximal device output that improved with generator and lead repositioning.^10^ Alternate electrode positioning was tried in only one patient in our cohort with a BMI > 40 kg/m^2^ who failed to respond to maximum shocks with a left parasternal electrode. The electrode was moved to the right parasternal location during the index procedure without a change in defibrillation success. This was similar to the findings of Levine et al., who noted a lack of DFT reduction with coil and generator repositioning in a patient with a BMI > 40 kg/m^2^. In this case report, a transvenous system was eventually implanted, with demonstration of adequate DFT.^11^ Guenther et al. reported on their experience with electrode tunneling in a substernal, epicardial location, which resulted in successful defibrillation following failure to defibrillate with a conventional left parasternal position.^12^ In our cohort, two of the patients who initially failed shocks at 80 J achieved acceptable DFT following the initiation of oral sotalol and without any change in shock impedance. Sotalol has been demonstrated to lower the DFT in transvenous ICD defibrillation,^13^ and could have a similar benefit in scenarios of high DFT with the S-ICD.

A positive correlation between high BMI and high DFT was demonstrated in our study cohort, in particular for BMI values exceeding 35 kg/m^2^. As such, a BMI limit of 35 kg/m^2^ may serve as an important critical threshold above which defibrillation failure with the S-ICD is more likely to occur. The elevated DFT in obese patients could be related to the presence of increased adipose tissue, which is a poor electrical conductor. Heist et al. showed through computer modeling with the S-ICD that sub-coil adipose tissue increased the DFT significantly.^14^ Furthermore, DFT also increased when the S-ICD generator was placed interior to the optimal position. Among our patients, extensive efforts were made to implant and tunnel the S-ICD components within the fascial plane and below the subcutaneous fat. While generator positioning in the fascial space could be confirmed by direct visualization, electrode tunneling in the subcutaneous fat along the sternum may be more apt to occur in obese patients. Increased distance between the device and coil in patients with large chest circumferences, increased lung volumes, or increased anteroposterior chest diameter could also cause increased transthoracic resistance to shock current delivery, resulting in shock failure. Elevated transthoracic resistance has been strongly correlated with increased chest width and chest wall thickness.^15^ Failure to position the generator sufficiently lateral or posterior enough along the chest wall in patients with morbid obesity and very large chest circumferences may result in a system configuration that delivers an inadequate shock current to the ventricular myocardium.

Patients with significant renal functional impairment based on a glomerular filtration rate (GFR) < 30 mL/min were excluded from the early pivotal S-ICD study. In contrast, the incidence of ESRD on HD was 32.7% for our entire implant cohort, and 19% of all patients who underwent full DFT testing, respectively. There was no significant difference in DFT between subjects with and without ESRD. The theoretical advantages of the S-ICD are especially important in patients on HD given their increased risk for device-related infections and their limited vascular access when considering transvenous systems. Our study findings show that the S-ICD can be implanted safely in patients receiving HD with adequate defibrillation safety margin. The lower shock impedance seen in patients with ESRD could be attributed to a smaller BMI than in those without ESRD. In our study, ESRD patients tended to have lower BMIs (26.9 kg/m^2^ versus 31.7 kg/m^2^; p = 0.13), though this failed to reach statistical significance.

Our study noted that ventricular wall thicknesses predict elevated DFT with the S-ICD. While the LVEDD measured on echocardiogram has been shown to predict elevated DFT with transvenous systems, increased wall thickness, particularly of the interventricular septum and posterior wall, respectively, appeared to correlate with higher DFT values with the S-ICD. Mizukami et al. found that an interventricular septal thickness of more than 12 mm was an independent predictor for high DFT in transvenous ICD systems.^16^ Given that the shock vector of the S-ICD system is between the left lateral and anterior chest walls, it is plausible that insufficient energy delivery occurs with increased septal and posterior wall mass, resulting in a failure to defibrillate.

### Clinical implications

Our data suggest that in a contemporary population undergoing S-ICD implantation, elevated DFT may be more likely to be present in the morbidly obese population, particularly with BMI > 35 kg/m^2^ and in subjects with increased ventricular wall thicknesses in either the septal or posterior wall. Greater consideration of systematic DFT testing or type of defibrillator system in these patient groups is recommended based on our findings. Further investigation is needed to validate these risk factors. Conversely, our study also demonstrates the feasibility of defibrillating patients successfully with low energy outputs. Eleven out of 31 patients (35%) had DFT ≤ 25 J, and 65% of the tested cohort had DFT of ≤ 50 J. Predictable low shock requirements could encourage the development of programmable, tailored shock therapies (which could result in shorter charge times to shock delivery), or the development of smaller ICD generators in patients with predictably low DFTs.

### Areas for future investigation

Based on our findings, high failed shock impedance and left ventricular wall thickness may correlate with high DFTs. The ability to both measure and correlate a non-invasively obtained cutaneous chest wall impedance with the shock impedance obtained during testing at implant could provide the foundation for developing a pre-procedure screening tool that identifies subjects who may have high shock impedance values with the S-ICD and a higher risk of high DFT and defibrillation failure. In addition, such testing might also predict a low energy requirement in which testing may not be necessary, similar to contemporary transvenous ICD placement.

### Limitations

The present study is limited by its retrospective nature, relatively small sample size, and non-uniform defibrillation testing protocol. Still, our single-center experience with dedicated efforts to perform some degree of step-down or full DFT testing in as many patients as possible is among the largest studies evaluating step-down defibrillation testing with the S-ICD. It is important to note though that our total cohort size did not permit adequate subgroup analyses. In addition, given that DFT testing is probabilistic in nature, the lack of repetitive confirmatory testing decreases its accuracy.

## Conclusions

Systematic step-down defibrillation testing in a heterogeneous patient population receiving the S-ICD, including morbidly obese patients and those with ESRD, shows a higher incidence of inadequate DFT safety margin with the S-ICD than originally reported in early device experience. Morbid obesity with BMI ≥ 35 kg/m^2^ higher BSA, and increased septal and posterior wall thickness may predict higher DFT and an increased risk of defibrillation failure with the S-ICD. Additional studies with larger sample sizes are needed to further characterize the potential risk factors for unacceptably high DFTs.

## Figures and Tables

**Table 1:** Baseline Demographic Data of All Patients Who Underwent S-ICD DFT Testing

	Population (n = 50)
**Demographics, comorbidities, and medications**	
Age (years)	50.9±13.8
Male gender (%)	37 (74%)
Body mass index (kg/m^2^)	29.3±6.8
Body surface area (m^2^)	2.0±0.3
Ischemic (%)	21 (42%)
Non-ischemic (%)	29 (58%)
End-stage renal disease (%)	16 (32%)
Hypertension (%)	26 (52%)
Diabetes mellitus (%)	17 (34%)
ACEI/ARB (%)	31 (62%)
Aldactone (%)	14 (28%)
Antiarrhythmic (%)	10 (20%)
**Indication**	
Primary (%)	36 (72%)
Secondary (%)	14 (28%)
Old device infection (%)	4 (8%)
ECG parameters	
Heart rate (beats/min)	70.4±13.5
QRS duration (ms)	112.3±19.3
Left ventricular hypertrophy (%)	9 (18%)
**Echocardiographic parameters**	
Ejection fraction (%)	31.4±13.4
Septal thickness (cm)	1.1±0.3
Posterior wall thickness (cm)	1.1±0.3
LVEDD (cm)	5.8±1.0
Effusion (%)	6 (12%)
**Laboratory values**	
Blood urea nitrogen (mg/dL)	29.2±19.4
Creatinine (mg/dL)	2.9±2.9
Sodium (mmol/L)	138.1±3.4
Potassium (mmol/L)	4.4±0.6
Magnesium (mmol/L)	2.1±0.3
**DFT data (n = 50)**	
Average number of shocks (times)	3.9±2.1
Average success shock impedance (Ω)	67.3±25.2
Average failed shock impedance (Ω)	82.5±39.4
Fluoroscopy (min)	0.89±0.9
DFT (J)	50.7±26.3

S-ICD: subcutaneous implantable cardioverter-defibrillator; DFT: defibrillation threshold; ACEI: angiotensin-converting enzyme inhibitor; ARB: angiotensin receptor blocker; ECG: electrocardiogram; LVEDD: left ventricular end-diastolic dimension; J: joules.

**Table 2:** A Comparison of the High and Normal DFT Groups

	Normal DFT ≤ 65 J (n = 26)	High DFT > 65 J (n = 5)	p
**Demographics, comorbidities, and medications Age (years)**			
Age (years)	52.0±11.6	44.0±15.2	0.19
BMI (kg/m^2^)	29.4±6.1	37.7±7.1	0.02
Obese (BMI ≥ 30)	13 (50.0%)	5 (100.0%)	0.06
Obese (BMI ≥ 35)	3 (11.5%)	3 (60.0%)	0.04
Body surface area (m^2^)	1.9±0.3	2.4±0.1	0.0002
Ischemic (%)	11 (42.3%)	1 (20.0%)	0.62
Non-ischemic (%)	15 (57.7%)	4 (80.0%)	0.62
β-blocker (%)	18 (69.2%)	5 (100.0%)	0.15
ACEI/ARB (%)	16 (61.5%)	3 (60.0%)	1
Aldactone (%)	9 (34.6%)	3 (60.0%)	0.35
Amiodarone (%)	6 (23.1%)	1 (20.0%)	1
Hypertension (%)	13 (50.0%)	3 (60.0%)	1
Diabetes mellitus (%)	9 (34.6%)	2 (40.0%)	1
End-stage renal disease (%)	5 (19.2%)	1 (20.0%)	1
**Indication**			
Primary (%)	13 (50.0%)	5 (100.0%)	0.06
Secondary (%)	13 (50.0%)	0 (0%)	0.06
Infection (%)	3 (11.5%)	0 (0%)	1
**ECG and echocardiographic parameters**			
Heart rate (beats/min)	69.5±12.3	74.6±21.3	0.46
QRS duration (ms)	114.3±18.7	107.6±13.0	0.45
center ventricular hypertrophy (%)	6 (23.1%)	0 (0%)	0.55
Ejection fraction (%)	33.6±15.1	26.0±7.4	0.28
Septal thickness (cm)	1.0±0.2	1.5±0.2	0.0003
Posterior wall thickness	1.1±0.2	1.4±0.3	0.003
LVEDD (cm)	5.6±1.0	6.2±0.5	0.19
Effusion (%)	2 (7.7%)	0 (0%)	1
**Laboratory values**			
Blood urea nitrogen (mg/dL)	26.2±20.0	20.2±6.8	0.52
Creatinine (mg/dL)	2.2±2.3	2.0±2.6	0.85
Sodium (mmol/L)	138.3±3.1	138.6±2.6	0.83
Potassium (mmol/L)	4.3±0.5	4.3±0.4	0.96
Magnesium (mmol/L)	2.0±0.3	2.1±0.1	0.73
**DFT data (n = 50)**			
Average number of shocks (times)	4.7±1.2	5.8±3.8	0.23
Average success shock impedance (Ω)	73.3±26.8	78.7±29.5	0.005
Average failed shock impedance (Ω)	71.5±26.4	138.0±55.8	0.76
DFT (J)	38.5±19.4	88.0±10.9	0.0005

DFT: defibrillation threshold; J: joules; BMI: body mass index; ACEI: angiotensin-converting enzyme inhibitor; ARB: angiotensin receptor blocker; ECG: electrocardiogram; LVEDD: left ventricular end-diastolic dimension.

**Table 3:** A Comparison of ECG and Echocardiographic Values of the Normal and High DFT Groups

	Normal DFT ≤ 65 J (n = 26)	High DFT > 65 J (n = 5)	p
Ejection fraction (%)	33.6±15.1	26.0±7.4	0.28
Septal thickness (cm)	1.0±0.2	1.5±0.2	0.0003
Posterior wall thickness (cm)	1.1±0.2	1.4±0.3	0.003
LVEDD (cm)	5.6±1.0	6.2±0.5	0.19
Pericardial effusion (%)	2 (7.7%)	0 (0%)	1
Heart Rate (beats/min)	69.5±12.3	74.6±21.3	0.46
QRS duration (ms)	114.3±18.7	107.6±13.0	0.45
center ventricular hypertrophy (%)	6 (23.1%)	0 (0%)	0.55
Lead I (mV)	7.7±5.4	8.0±3.2	0.9
Lead aVL (mV)	7.2±6.1	6.4±2.8	0.77
V5 (mV)	12.4±7.4	10.2±4.8	0.53
V6 (mV)	11.2±6.6	9.8±4.4	0.64

ECG: electrocardiogram; DFT: defibrillation threshold; J: joules; LVEDD: LVEDD: left ventricular end-diastolic dimension.

**Table 4:** A Comparison of Study Subjects with and without ESRD

	No ESRD (n = 25)	ESRD (n = 6)	p
**Demographics, comorbidities, and medications**			
Age (years)	50.2±13.3	52.8±7.8	0.65
BMI (kg/m^2^)	31.7±6.8	26.9±6.3	0.13
BSA (m^2^)	2.0±0.3	1.9±0.4	0.18
Ischemic (%)	7 (28.0%)	5 (83.3%)	0.02
Non-ischemic (%)	18 (72.0%)	1 (16.7%)	0.02
β-blocker (%)	18 (72.0%)	5 (83.3%)	1
ACEI/ARB (%)	16 (64.0%)	3 (50.0%)	0.65
Aldactone (%)	12 (48.0%)	0 (0%)	0.06
Amiodarone (%)	4 (16.0%)	3 (50.0%)	0.11
Hypertension (%)	12 (48.0%)	4 (66.7%)	0.65
Diabetes mellitus (%)	7 (28.0%)	4 (66.7%)	0.15
**Indications**			
Primary (%)	14 (56.0%)	4 (66.7%)	1
Secondary (%)	11 (44.0%)	2 (33.3%)	1
Infection (%)	3 (12.0%)	0 (0%)	1
**ECG and echocardiographic parameters**			
Heart rate (beats/min)	68.5±13.3	78.2±14.0	0.12
QRS duration (ms)	112.8±17.1	115.0±22.8	0.79
Left ventricular hypertrophy (%)	5 (20.0%)	1 (16.7%)	1
Ejection fraction (%)	33.0±15.4	29.8±8.9	0.64
Septal thickness (cm)	1.1±0.2	1.1±0.3	0.84
Posterior wall thickness (cm)	1.1±0.3	1.2±0.3	0.46
LVEDD (cm)	5.7±1.0	5.8±0.5	0.88
Effusion (%)	2 (8.0%)	0 (0%)	1
**Laboratory values**			
Blood urea nitrogen (mg/dL)	21.2±16.3	41.7±19.9	0.01
Creatinine (mg/dL)	1.1±0.5	6.5±1.2	o0.0001
Sodium (mmol/L)	138.7±3.0	136.7±2.7	0.14
Potassium (mmol/L)	4.3±0.5	4.4±0.5	0.76
Magnesium (mmol/L)	1.9±0.2	2.4±0.3	0.001
**DFT data**			
Average number of shocks	4.8±1.9	5.3±1.4	0.52
Average success shock impedance (Ω)	82.9±26.2	55.2±15.7	0.03
Average failed shock impedance (Ω)	97.9±42.8	56.3±15.3	0.04
DFT (J)	51.4±30.3	51.7±34.7	0.99

ESRD: end-stage renal disease; ACEI: angiotensin-converting enzyme inhibitor; ARB: angiotensin receptor blocker; DFT: defibrillation threshold; ECG: electrocardiogram; LVEDD: left ventricular end-diastolic dimension.

**Table 5:** Univariate Regression Analysis of Predictors for High DFT in 31 Patients Who Underwent Full DFT Testing

Variable	High DFT (n = 5)		
	Odds Ratio	95% CI	p
Age (years)	0.94 (n = 5)	0.86–1.03	0.19
Ejection fraction	0.95 (n = 5)	0.87–1.04	0.28
Heart rate	1.03 (n = 5)	0.96–1.1	0.45
QRS duration	0.98 (n = 5)	0.92–1.04	0.44
BMI (per 5 units)	1.2 (n = 5)	1.02–1.41	0.03
Underweight (BMI < 18.5)	0.29 (n = 0)	0.01–7.41	0.45
Normal (18.5 ≤ BMI ≤ 24.9)	0.29 (n = 0)	0.01–7.41	0.45
Overweight (25 ≤ BMI ≤ 29.9)	0.29 (n = 0)	0.01–7.41	0.45
Obese (BMI ≥ 30)	11.0 (n = 5)	0.50–244.46	0.13
Obese (BMI ≥ 35)	11.5 (n = 3)	1.33–99.34	0.03
Body surface area (m^2^)	>999.99 (n = 5)	5.53–>999.99	0.02
Ischemic	0.34 (n = 1)	0.03–3.49	0.36
Non–ischemic	3.43 (n = 4)	0.34–34.98	0.3
β–Blocker	5.50 (n = 5)	0.21–121.19	0.32
ACEI/ARB	0.94 (n = 3)	0.13–6.63	0.95
Aldactone	2.83 (n = 3)	0.4–20.18	0.3
Amiodarone	0.83 (n = 1)	0.08–8.95	0.88
End–stage renal disease	1.05 (n = 1)	0.1–11.56	0.97
Primary indication	11.0 (n = 5)	0.50–244.46	0.13
Secondary indication	0.24 (n = 0)	0.01–5.86	0.38
Device infection indication	0.61 (n = 0)	0.02–21.24	0.79
Hypertension	1.5 (n = 3)	0.21–10.51	0.68
Diabetes mellitus	1.26 (n = 2)	0.18–8.97	0.82
Lead I	1.01 (n = 5)	0.84–1.22	0.89
Lead aVL	0.97 (n = 5)	0.78–1.2	0.76
V5	0.95 (n = 5)	0.82–1.11	0.52
V6	0.96 (n = 5)	0.81–1.14	0.62
LVH by ECG	0.27 (n = 0)	0.01–7.06	0.43
Septal thickness	596.91 (n = 5)	4.03–>999.99	0.01
Posterior wall thickness	234.34 (n = 5)	2.08–>999.99	0.02
LVEDD	1.91 (n = 5)	0.72–5.03	0.19
Effusion	0.89 (n = 0)	0.02–41.28	0.95
Blood urea nitrogen (mg/dL)	0.98 (n = 5)	0.91–1.05	0.52
Creatinine (mg/dL)	0.95 (n = 5)	0.6–1.51	0.84
Sodium (mmol/L)	1.04 (n = 5)	0.75–1.44	0.82
Potassium (mmol/L)	1.05 (n = 5)	0.15–7.31	0.96
Magnesium (mmol/L)	2.15 (n = 5)	0.03–144.8	0.72
Average number of shocks	1.32 (n = 5)	0.82–2.15	0.25
Average success shock impedance (Ω)	1.01 (n = 3)	0.96–1.06	0.74
Average failed shock impedance (Ω)	1.05 (n = 3)	1–1.1	0.05

DFT: defibrillation threshold; CI: confidence interval; BMI: body mass index; ACEI: angiotensin-converting enzyme inhibitor; ARB: angiotensin receptor blocker; LVH: left ventricular hypertrophy; ECG: electrocardiogram; LVEDD: left ventricular end-diastolic dimension
